# Collective Vortex-Like Movement of *Bacillus subtilis* Facilitates the Generation of Floating Biofilms

**DOI:** 10.3389/fmicb.2018.00590

**Published:** 2018-03-29

**Authors:** Nitai Steinberg, Gili Rosenberg, Alona Keren-Paz, Ilana Kolodkin-Gal

**Affiliations:** Department of Molecular Genetics, Weizmann Institute of Science, Rehovot, Israel

**Keywords:** pellicles, biofilms, collective behavior, cell-cell communication, flagellar motility

## Abstract

Bacteria in nature are usually found in complex multicellular structures, called biofilms. One common form of a biofilm is *pellicle*—a floating mat of bacteria formed in the water-air interphase. So far, our knowledge on the basic mechanisms underlying the formation of biofilms at air-liquid interfaces is not complete. In particular, the co-occurrence of motile cells and extracellular matrix producers has not been studied. In addition, the potential involvement of chemical communication in pellicle formation remained largely undefined. Our results indicate that vortex-like collective motility by aggregates of motile cells and EPS producers accelerate the formation of floating biofilms. Successful aggregation and migration to the water-air interphase depend on the chemical communication signal autoinducer 2 (AI-2). This ability of bacteria to form a biofilm in a preferable niche ahead of their potential rivals would provide a fitness advantage in the context of inter-species competition.

## Introduction

Bacteria in nature are most often found in the form of multicellular communities known as biofilms (Kolter and Greenberg, 2006; Aguilar et al., 2007). When compared to planktonic (free-living) state, cells in biofilms attach more firmly to hosts, have better access to nutrients, and are more protected from environmental insults, including sterilizing agents, antibiotics, and the immune system, (Stewart, 1994, 2002; Costerton et al., 1999; Stewart and Costerton, 2001; Donlan and Costerton, 2002; Mah et al., 2003; Fux et al., 2005).

The model organism *Bacillus subtilis* is a genetically manipulatable robust biofilm former that resides in soil, where it colonizes plant roots and protects its hosts from fungi and other bacterial species (Vlamakis et al., 2013). A subpopulation of flagellated cells (Kearns and Losick, 2005) was demonstrated to exist both in *B. subtilis* floating biofilms, and in biofilms formed on the solid surfaces (Vlamakis et al., 2008). *B. subtilis* uses its' peritrichous flagella for both swimming and swarming (Mukherjee and Kearns, 2014). Similar to other bacterial flagella, *B. subtilis* flagella consists of a filament composed of subunits of the flagellin protein, encoded by the gene *hag* (LaVallie and Stahl, 1989), and a motor subunit that enables the flagellar hook and filament rotation, formed by the proteins MotA and MotB (Mirel et al., 1992). The direction of rotation of the flagella, and consequently the mode of swimming, is influenced by the chemotaxis machinery, which is governed by the two component system CheA-CheY (Mukherjee and Kearns, 2014). The flagellar genes are tightly regulated, and expressed only under specific conditions (Guttenplan et al., 2013).

In *B. subtilis* biofilms, motile cells are considered a minority (Vlamakis et al., 2008), while the majority of the cells are held together by the exopolymeric substances (EPS) matrix. The EPS is composed of exopolysaccharides synthesized by the products of the *eps*A-O operon, the BslA protein that forms a hydrophobic coat, and TasA—a functional amyloid (Vlamakis et al., 2013). The EPS microenvironment was suggested to limit flagellar motility (Cairns et al., 2013). However, EPS may also promote collective motility, by generating groups of motile cells.

We here explored the potential roles of flagellated cells and collective motility in floating biofilms. Natural isolates of *B. subtilis* are a robust model for pellicle formation, as they are capable of forming highly structured biofilms in the interphase between the liquid medium and the air (Mcloon et al., 2011). Motility, EPS production and chemotaxis were shown to contribute to the efficiency of pellicle formation (Branda et al., 2004; Holscher et al., 2015). Furthermore, in a genetic screen, it was shown that both motility and EPS promote pellicle formation together in its early stages (Kobayashi, 2007). This raises the possibility that migration of the biofilm cells to the water-air interphase may be collective, and rely on communication and collaboration between the EPS producers and motile cells.

To test this hypothesis, we follow the pellicle formation in *B. subtilis* under various conditions in real time, and analyze the potential contribution of motility genes to the dynamics of pellicle formation. We show that aggregates of EPS producers and motile cells are formed during pellicle formation. We demonstrate that these aggregates can exhibit vortex-like collective motion in liquid, and contribute to biofilm formation in the water-air interphase. Finally, we propose a mechanism that allows the formation of these large groups.

## Materials and methods

### Bacterial strains and strain construction

All experiments were performed with *B. subtilis* NCIB 3610 (Branda et al., 2001). Laboratory strains of *B. subtilis* (PY79) were used for cloning purposes. List of strains is provided in Table S1. Transformation of *B. subtilis* PY79 with PCR products was performed as previously described (Wilson and Bott, 1968). Transformation of NCIB 3610 was performed as previously described (Kolodkin-Gal et al., 2007; Yuan et al., 2011). Briefly, PCR products were first transformed into PY79, and then the genomic DNA of the transformed strain was transformed to NCIB 3610. Deletion mutations were generated by long-flanking homology PCR mutagenesis (Wach, 1996), primers are listed in Table S2.

### Media

The strains were routinely manipulated in LB broth (Difco, Falcon BD, Corning, NY, USA) or MSgg medium (5 mM potassium phosphate, 100 mM MOPS pH 7, 2 mM MgCl2, 50 μM MnCl_2_, 50 μM FeCl_3_, 700 μM CaCl_2_, 1 μM ZnCl_2_, 50 μM FeCl_3._ 2 μM thiamine, 0.5% glycerol, 0.5% glutamate, 50 μg ml^−1^ threonine, tryptophan and phenylalanine; Branda et al., 2001).

Selective media for cloning purposes were prepared with LB or LB-agar using antibiotics at the following final concentrations: 100 μg ml^−1^ ampicillin, 10 μg ml^−1^ kanamycin from AG Scientific (San-Diego, California, USA), 10 μg ml^−1^ chloramphenicol, 10 μg ml^−1^ tetracycline, 100 μg ml^−1^ spectinomycin from Amersco (Dallas, Texas, USA) and 1 μg ml^−1^ erythromycin (Amresco, Dallas, Texas, USA) + 25 μg ml^−1^ lincomycin (Sigma Aldrich, St. Louis, Missouri, USA for MLS.

### Floating biofilms development assay

Cells were grown in LB from a single colony isolated over LB plates to a mid-logarithmic phase (4 h at 37°C with shaking). For floating biofilm assays, the inoculum ratio was 1:1,000. Pellicle phenotypes in cultures grown at 23°C were observed. Photos of floating biofilms were acquired with a NikonD800 camera (Nikon, Tokyo, Japan) or a stereomicroscope (Zeiss, Oberkochenm, Germany) and images were optimized for contrast and brightness, with adjustments kept consistent. For evaluation of the effect of the surface area on pellicle formation, cells were re-diluted 1:1,000 in liquid MSgg medium in cell-culture multiwell plates (Thermo Scientific, Waltham, Massachusetts, USA) of 48, 24, 12, or 6 wells, containing a total volume of 0.85, 1.58, 2.95, or 8.6 ml of medium per-well, respectively, in order to reach comparable height without shaking. Plates were incubated at room temperature.

### Floating biofilm kinetics assays

**Figure 1:**
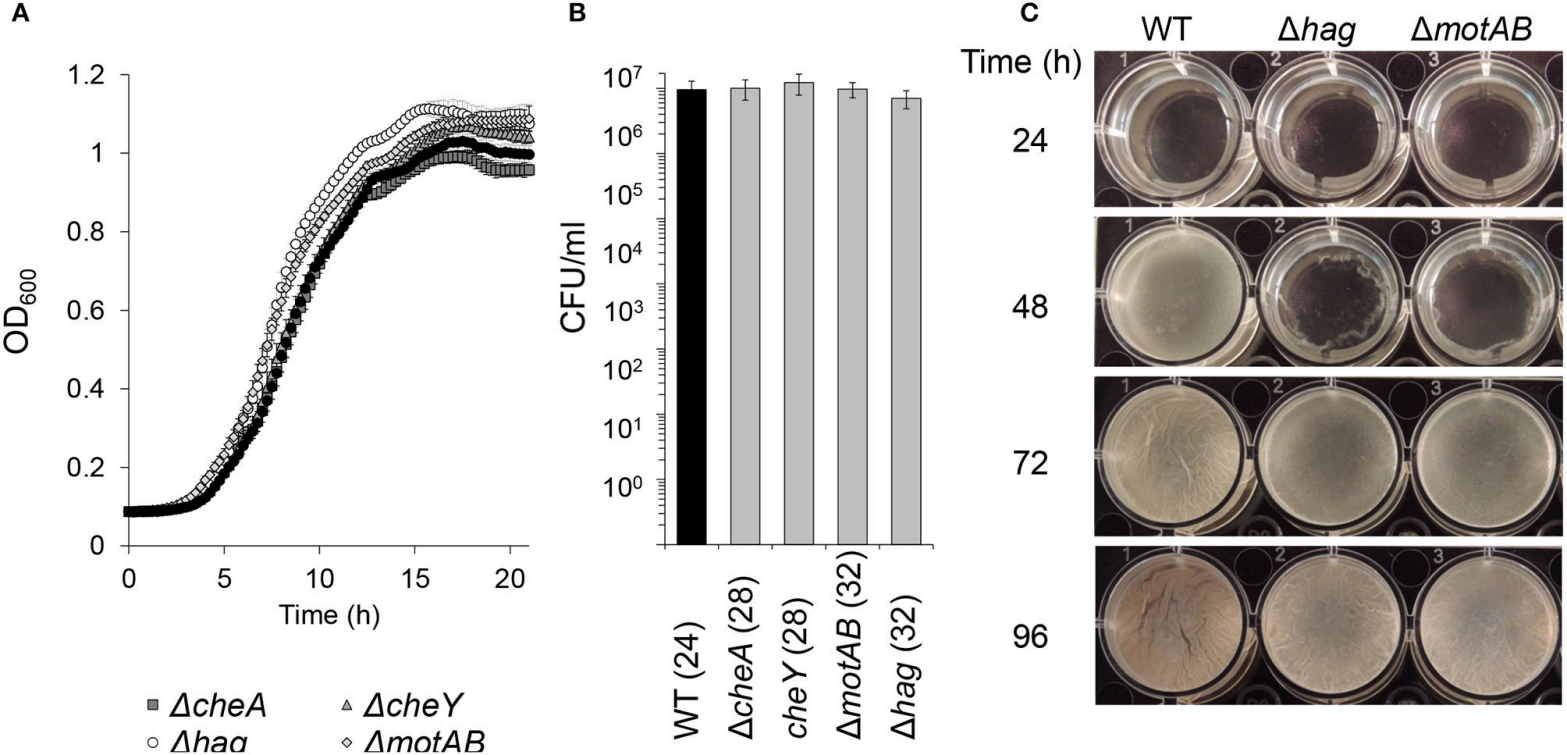
The effects of flagellar motility and chemotaxis on planktonic growth and floating biofilm formation. **(A)** Growth curves of wild-type and motility and chemotaxis mutants in shaking liquid cultures in biofilm inducing medium (MSgg). Error bars represent standard deviation. **(B)** Live cell counts of bacteria grown in standing liquid cultures before the floating biofilm is formed. Cultures were grown and harvested for CFU analyses (time post-inoculation is indicated in parenthesis). The differences between the wild-type and the indicated motility mutants were found as insignificant (*p* > 0.5) in a two tailed paired student's *t*-test vs. the wild-type. **(C)** Images of pellicle formation process in wild-type and motility mutants in standing liquid cultures at 23°C.

**Figure 2:**
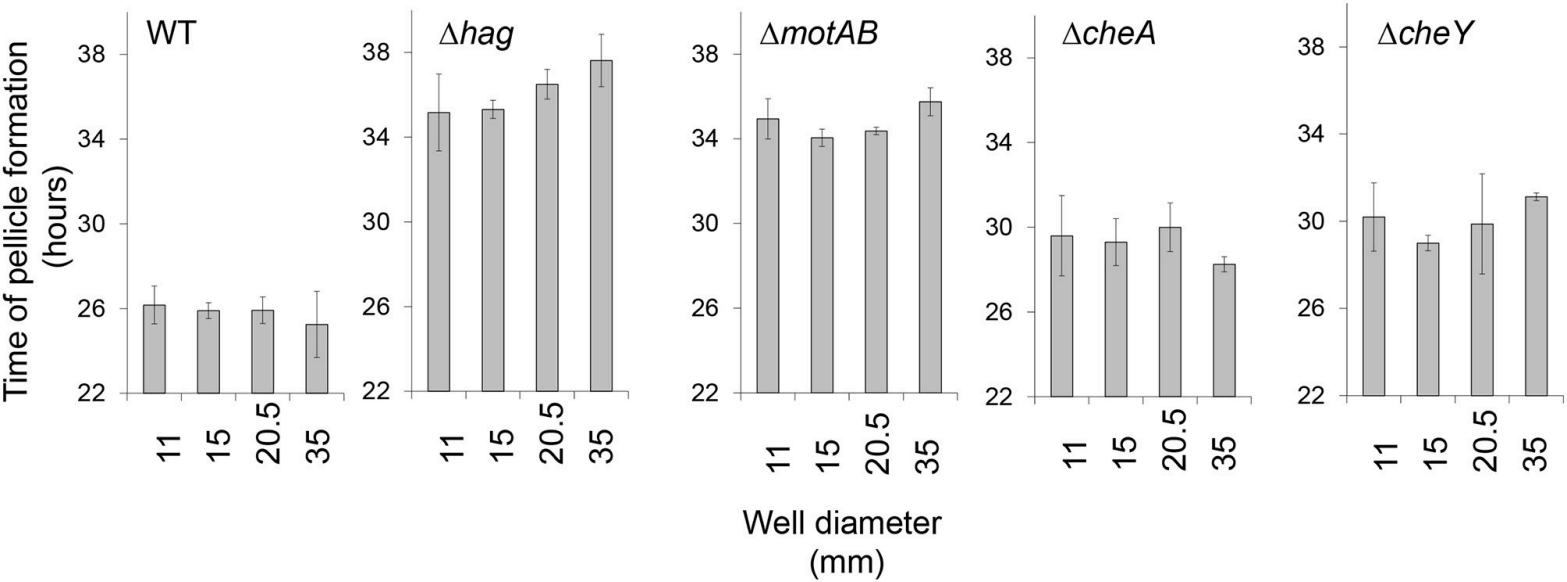
The impact of surface area on floating biofilm formation. Floating biofilm formation time of wild-type and motility and chemotaxis mutants in standing liquid cultures was monitored using a Nikon D800 camera (Nikon, Tokyo, Japan) and images were analyzed manually in order to identify time point of floating biofilm formation, for details refer to Materials and Methods. Error bars represent standard deviation. The effect of an increase of well diameter on the delay of pellicle formation was found insignificant within each given background (*P* > 0.3). The Wild-type strain differed significantly from all motility mutants for each condition (*P* < 0.05).

**Figure 3:**
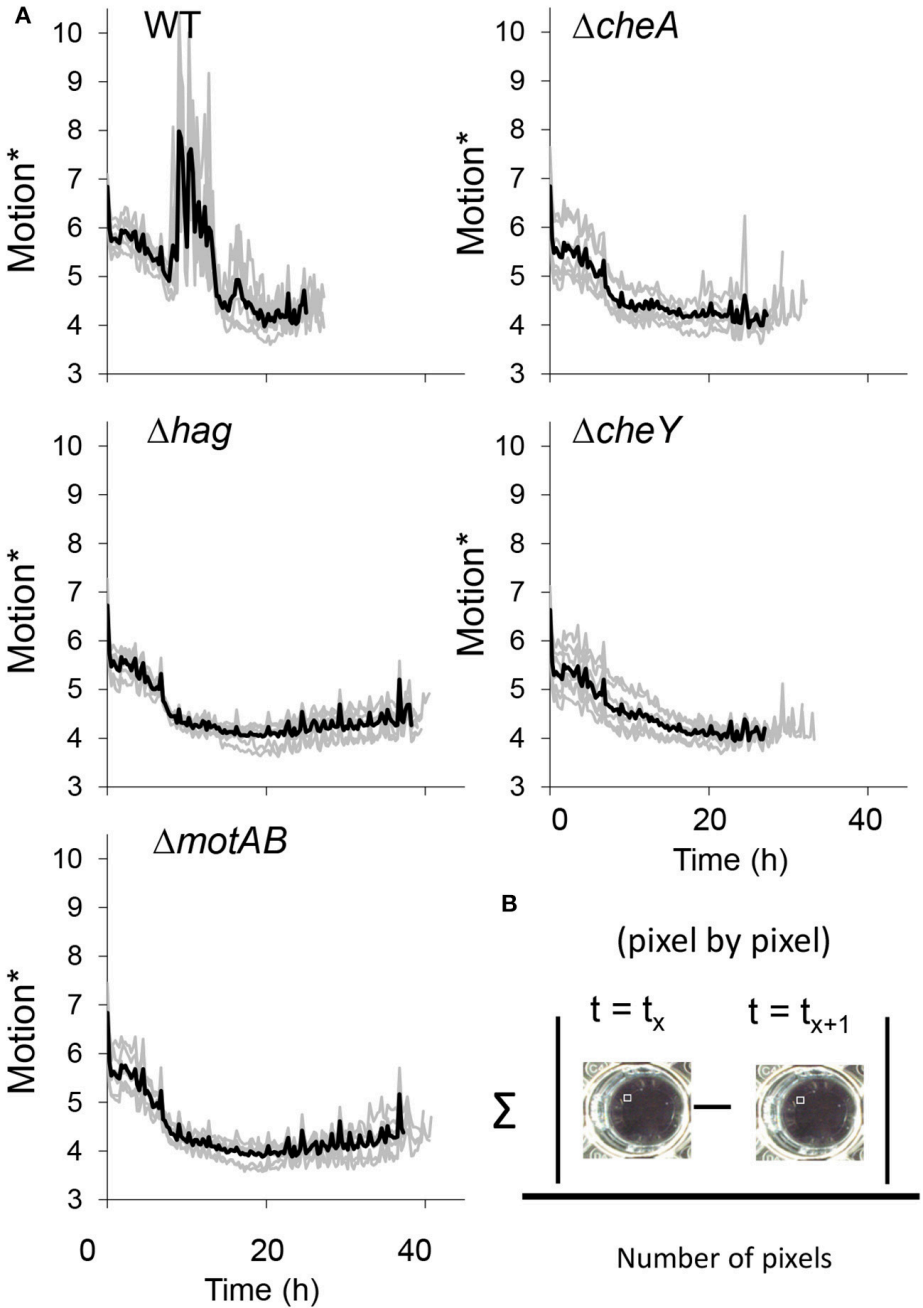
**(A)** The collective motion during floating biofilm formation. Motion^*^ was defined as the mean of absolute difference between pixels of each two consecutive frames was calculated in time-lapse images series taken using a Nikon D800 camera of standing liquid cultures during floating biofilm formation, as described in Materials and Methods. **(B)** Darker thick line—means of seven technical repeats for each strain, lighter thin line—the value for each of the repeats. See also Videos S1, S2.

For Floating biofilm kinetics assays (Figures 2, 3), Cells were grown as described above for floating biofilm development essay. Photo images were taken every 15 min using a Nikon D800 camera (Nikon, Tokyo, Japan) for up to 40 h. Images were analyzed manually in order to identify time point of pellicle formation. Quantification of motion was performed using a custom Matlab code. Briefly, the mean of absolute values of the differences between each pixel in a given frame and its matching pixel in the consecutive frame was calculated as a measure of motion in a given time point. In order to quantify this motion, we used the mean of absolute differences between pixels of consecutive frames (of the top-view images) as a measure of the motion during the specific time-lapse (Figures 3A,B). The MatLab script is included as Datasheet 1. Note that time-lapse images were taken at room temperature and therefore there was a minor shift between the images shown in Figure 1C and time-lapse image analysis provided in Figure 2 and Video S1, however, the trend was the same.

### Planktonic growth measurements

Cells were grown from a single colony isolated over LB plates to a mid-logarithmic phase (4 h at 37°C with shaking). Cells were diluted 1:100 in 150 μl liquid MSgg medium of each well of a 96-well microplate Thermo Scientific, Waltham, Massachusetts, USA). Cells were grown with agitation at 30°C for 20 h in a Synergy 2 microplate reader (BioTek, Winooski, Vermont, United States), and the optical density at 600 nm (OD_600_) was measured every 15 min.

### Determination of cell density and live cell counts during floating biofilm formation

To determine culture density and live cell counts of cells grown in pellicles, cells were harvested from a floating biofilm assay (described above), and thoroughly vortexed. Untreated wild type cells were mildly sonicated at a BRANSON digital sonifier, Model 250, Microtip, with amplitude 20%, pulse 3 × 5 s (Thermo Scientific, Waltham, Massachusetts, USA). For culture density, OD_600_ was measured with a Ultrospec 2100 spectrophotometer (Amersham Biosciences, Little Chalfont, England). To determine the number of live cell counts, cells were serial diluted in phosphate-buffered saline (PBS) (Biological Industries, Israel), plated on LB-plates, and colony forming units (CFU) were counted after incubation at 30°C over-night.

### Fluorescence microscopy

**Figure 4:**
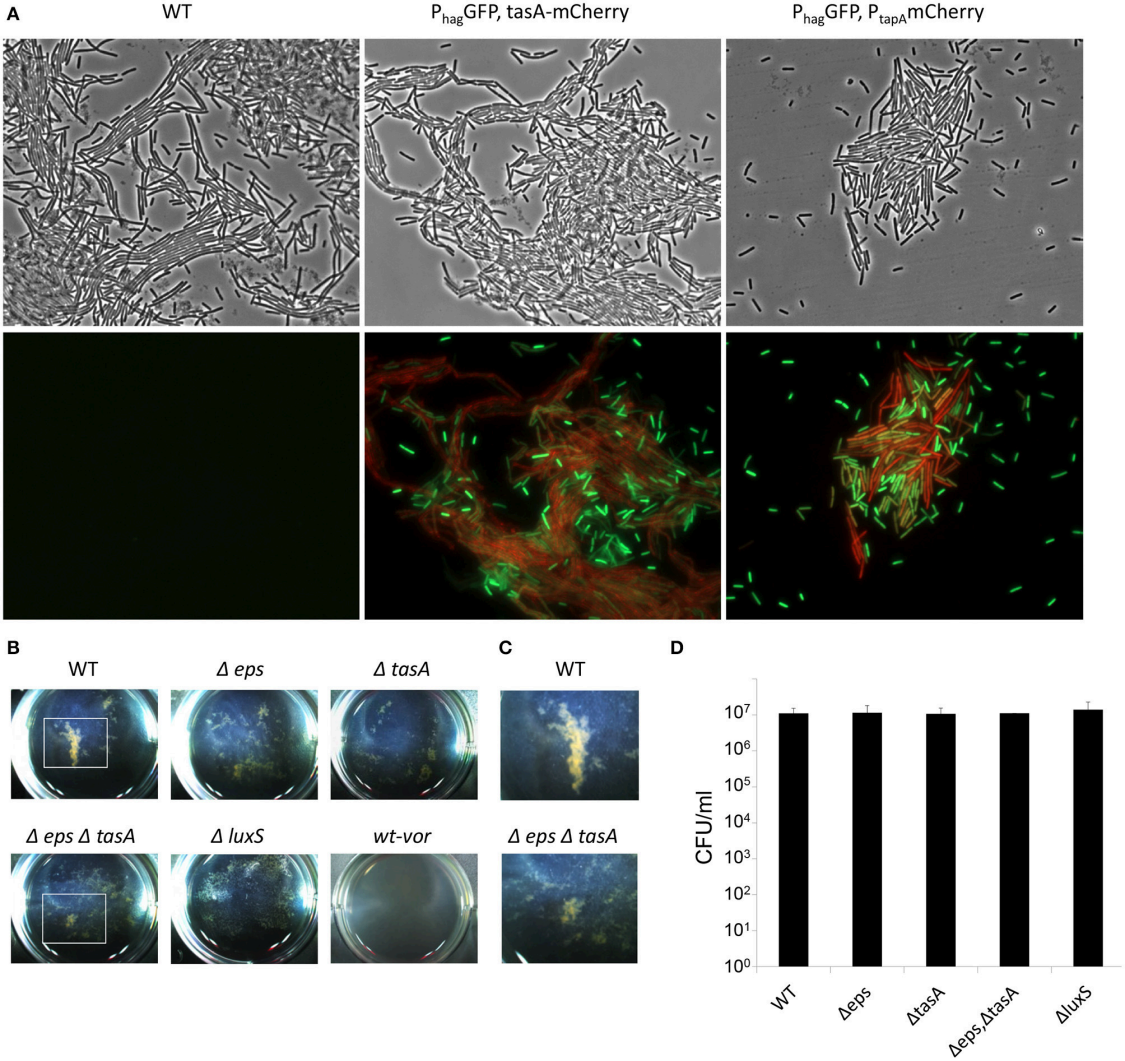
The aggregation of EPS producers and motile cells during floating biofilm formation. Bacteria were grown in standing liquid cultures in six well plates at 23°C for 20 h. **(A)** Wild-type and cells harboring P_*tapA*_*-mkateII* and P_*hag*_*-gfp*; or P_*tasA*_*-mCherry* and P_*hag*_*-gfp*. Cells were visualized by fluorescence microscopy; upper panel—phase contrast, lower panel—signal from GFP (green) and mKate/mCherry (red). The experiment shown here is a representative of 2 independent experiments; each done in at least duplicate. **(B)** Images of pellicle formation process in wild-type and the indicated mutants. WT-vor parental wild-type strain was collected at 20 h, robustly vortexed and returned to the well. **(C)** Enlargement of a well area defined with a white square in **(B)** Images are representative of three independent experiments, performed in triplicates. **(D)** Live cell counts of bacteria grown in standing liquid cultures before the pellicle is formed. Cultures were grown and harvested for CFU analyses 20 h post-inoculation. The differences between the wild-type and the indicated mutants were found as insignificant (*p* > 0.5) in a two tailed paired student's *t*-test vs. the wild-type.

An aggregate of cells from standing cultures grown as described in the legend to Figure 4 were placed on a microscope slide and covered with a poly-L-Lysine (Sigma)-treated coverslip. Cells were visualized and photographed using an Axioplan2 microscope (Zeiss) equipped with a high resolution microscopy Axiocam camera, as required. Data were captured using Axiovision suite software (Zeiss, Oberkochenm, Germany).

### AI-2 complementation

Synthetic DPD (4,5-dihydroxy-2,3-pentanedione) was applied to the growth medium at 10 μM. For the application of conditioned medium, wild-type and its *luxS* mutant cells were grown in a standing culture for 20 h as described in Figure 1. The growth medium was collected, centrifuged for 5 min at 8,000 rpm, and filtered through 0.22 μm filters. Conditioned medium was concentrated 50 times using the SpeedVac concentrator and applied in physiological concentration (e.g., diluted 50 times) into a fresh growth medium.

### Statistical methods

All studies were performed in triplicates at least three separate and independent times. Data are expressed as average values ± standard deviations of the means. Parametric testing was performed after confirming that raw data were normally distributed. Data were analyzed by two tailed student's *t*-test, used to determine if the set of mutants and treatments vs. the wild-type untreated control are different from each other and *P*-values of less than 0.1 were considered significant.

## Results

In order to better elucidate the various roles of motility in the biofilm state of bacteria, we examined floating biofilm formation in liquid. Wild-type *B. subtilis* cells are able to create a floating biofilm (pellicles) when grown in a standing liquid culture. The ability to form a pellicle was compared between the wild-type strain, and mutants in different components of the motility (*hag* and *motAB*) or chemotaxis (*cheA* and *cheY*) machineries (Figure 1). In shaking liquid cultures, the wild-type and the mutants had comparable growth (Figure 1A). The carrying capacity (as judged by maximal optical density), the lag-time and the growth rate were comparable to the parental wild type strain both in 30°C (Figure 1A) and in room temperature (data not shown). Moreover, CFU numbers during the stages prior to pellicle formation were comparable between all strains (Figure 1B). Next, time-lapse photography was then used to follow pellicle formation in the different strains in standing liquid cultures. While all strains eventually created a pellicle, its formation in mutant strains was significantly delayed (Figure 1C and Video S1). The extent of the delay in both motility and chemotaxis mutants was unexpectedly robust, and changes in the well area did not significantly affect it (Figure 2 and Video S1). The fixed relative contribution of the motile cells and the lack of response to the changes in the well area indicate that flagellar motility has an additional role, unrelated to reaching the liquid-air interphase, independent of surface parameters.

Consistently with the hypothesis that cells migrate using flagellar motility during pellicle formation, we noticed the appearance of turbid areas in the well before the formation of the floating biofilm. Importantly, in the wells of the wild-type, but not in those of the mutant strains, these turbid areas constantly changed their location between frames, implying collective motility of the cells during the 15 min long time-lapse (Video S1). Time-lapse images of a side-view of the wells clearly showed vortex-like movements of the cells before the pellicle was formed in the wild-type, but not in the motility or chemotaxis mutants (Figure 3 and Video S2). Between 10 and 20 h post-inoculation, the wild-type strain showed high levels of motion that were not seen in the motility or chemotaxis mutants. As the mutants were delayed in pellicle formation, it is possible that this collective movement is involved in accelerating the pellicle formation process. While vortex-like movement of bacteria was previously shown (Mendelson, 1999), this is the first evidence for the presence of collective movement in static liquid and for its connection to biofilm formation in water-air interphase. The aggregates may achieve a collective movement by cooperation between motile cells and matrix producers, a mechanism which was already shown to promote sliding motility on solid surfaces (van Gestel et al., 2015; Kovacs, 2016; Dragos and Kovacs, 2017).

To further confirm the presence and composition of macro-cell clusters in the early stages of pellicle formation, and to explore the mechanism that allows these clusters to form, the patterns observed during early static growth in the biofilm medium were carefully analyzed. Indeed, within 20 h, clear macro-clusters appeared in each well (Figures 4A–C). The clusters were held together by extracellular matrix polymeric substances, as their size and abundance was reduced dramatically both in a *tasA* mutant, lacking the proteinous component of the matrix, in an *eps* mutant unable to produce exopolysaccharides (Figure 4B), and in a double mutant for both extracellular matrix polymeric substances (Figures 4B,C). All the mutants failed to form a structured mature pellicle (Figure S1). Furthermore, a microscopic analysis of the TasA matrix protein showed that these aggregates were formed after the production of the ECM, and contained numerous motile cells (Figure 4A), while the surrounding medium contained isolated motile cells. Aggregation did not result from differential cell numbers (Figure 4D).

**Figure 5:**
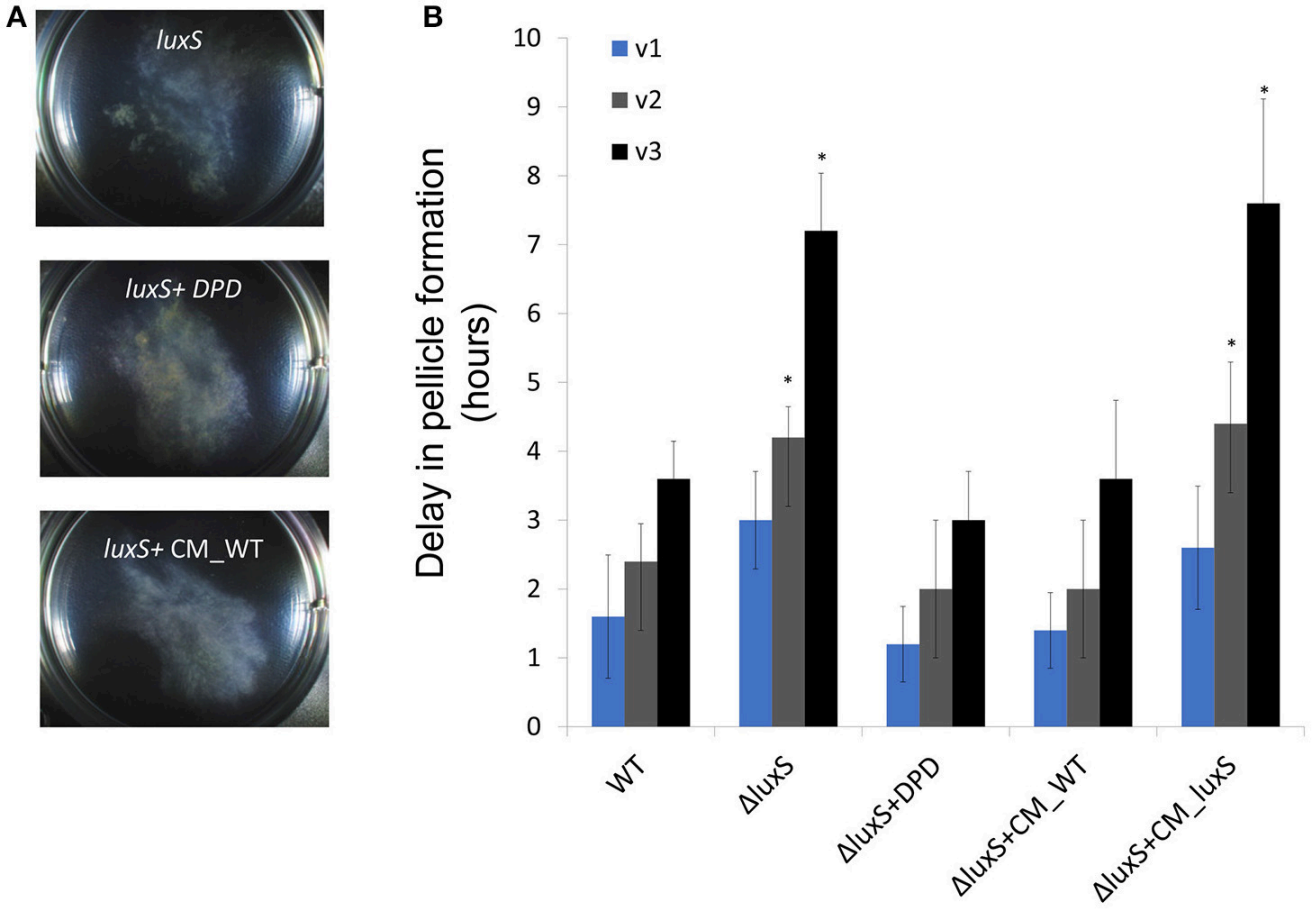
The requirement for aggregation during floating biofilm formation**. (A)** Top-down images of the aggregates within a six well plate plates at 23°C after 20 h of incubation. **(B)** At either 10 h (v1), or 10 and 16 h (v2), and 10, 16, and 20 h (v3) post-inoculation the growth medium was collected, robustly vortexed and returned to the well. The delay in floating biofilm formation was the difference between un-vortexed controls within the same experiment, and vortexed samples. Conditioned media was harbored from WT (CM_WT) and *luxS* mutant (CM_Δ*luxS*) floating biofilms grown under the same conditions and applied when indicated. Results are an average and standard deviation of 3 independent repeats performed in duplicates (^*^P ≤ 0.05).

Cell to cell communication may promote aggregation. The production of autoinducer-2 (AI-2) signal molecules, mediated by the activity of the LuxS enzyme, in response to changes in cell density, was established in *B. subtilis* (Lombardia et al., 2006). This autoinducer has been shown to promote initial biofilm formation and cell aggregation in several Gram-positive bacterial species (Lombardia et al., 2006; Trappetti et al., 2011; He et al., 2015). Consistently, we found that a mutant defective in AI-2 signaling is dramatically impaired in early aggregation, as well as in pellicle formation, indicating a role of this signal molecule in the formation of floating biofilms by *B. subtilis* (Figure 5). The aggregation of a *luxS* mutant was restored in the presence of DPD (4,5-dihydroxy-2,3-pentanedione), the precursor of AI-2, in agreement with previous studies (Miller and Bassler, 2001; Bassler and Losick, 2006; Camilli and Bassler, 2006; Ng and Bassler, 2009; Figure 5A). In contrast, mutants in flagellar motility formed macro-clusters similarly to the wild-type (Figure S2), suggesting that the formation of the clusters depends on extracellular matrix and cell-cell communication, whereas their collective motion appears to depend on flagellar motility. These results indicated that EPS matrix and chemical communication play a role in aggregation.

Lastly, we wanted to confirm that the collective motion has a role in promoting biofilm formation. To test this hypothesis, we disrupted the macro-clusters during early stages of pellicle formation. This disruption caused a reproducible delay in pellicle formation, without impairing the number of viable cells (Figure 5B). The delay was especially pronounced in a mutant lacking the AI-2 communication signals (Figure 5B). As mutants in exopolysaccharides do not form an established pellicle (Branda et al., 2004), it was impossible to assess whether they can recover from the dissolution. These results are consistent with the hypothesis that cell to cell communication mediated by the AI-2 pheromone has critical importance in the stabilization of macro-clusters, consistent with the complete restoration of aggregation properties by exogenous AI-2 (Figures 5A,B, Table S3). Similarly, a matrix mutant in *tasA* had a defect in re-aggregation following physical disturbance of the aggregates (Figure S3).

In this study, we sought to gain better understanding of the role of motility in bacterial floating biofilms. Motile cells enable collective motion in the early stages of floating biofilm formation. This motion can be seen in wild-type strain, but is absent in the mutant strains. Furthermore, collective motility accelerates pellicle formation (Figures 2, 3 and Videos S1, S2). Both flagellar rotation and chemotaxis contribute to pellicle formation under different conditions, suggesting the demand for flagellar motility is robust. Furthermore, it was demonstrated that the collective motility was made possible by vortex-like motion (Videos S1, S2), which is absent in the motility/chemotaxis mutants. Most the location of the air–liquid interface by sensing oxygen gradient, as previously shown (Holscher et al., 2015). However, cells can either first adhere randomly to the water-air interphase as single cells to generate a pellicle as suggested previously (Holscher et al., 2015), or collectively and directionally migrate to the interphase. Our results show collective translocation of bacterial vortices toward this niche.

The collective translocation to the interphase involves the formation of multicellular aggregates and strongly depends on diffusible signal molecule, AI-2 (Figure 5). This universal signal molecule regulates cell density-dependent phenotypes in many bacterial species (Ng and Bassler, 2009), and was previously shown to promote aggregation in Gram-positive pathogens (Lombardia et al., 2006; Trappetti et al., 2011; He et al., 2015). Single-cell analysis of the aggregating cell clusters indicates that they are primarily composed of EPS matrix producers and motile cells. As both EPS and rotated flagella are required for this motion, it is reminiscent of swarming motility, a rapid multicellular bacterial surface movement powered by rotating flagella, over solid surfaces (Kearns, 2010). Interestingly, *B. subtilis* does not make vortices during swarming on solid (Kearns, 2010), but exhibit vortex-like motion under our conditions in liquid (Videos S1, S2). These results may suggest a need for large, localized groups of cells traveling in a common circular path (generating a “vortex”) in liquid, and a potential need of the founders of the pellicle to act as a group to assemble the biofilm efficiently. These vortices are achieved by cooperation and chemical communication between two distinct cell types, EPS producers and motile cells.

In nature, bacteria reside in complex communities and an efficient colonization of a preferred growth niche can provide a significant advantage over potential competitors (Elias and Banin, 2012). We suggest that the robust and efficient strategy for enhanced colonization of interphases by *B. subtilis* may provide an essential mechanism for its survival in complex multispecies environments.

## Author contributions

NS, GR, AK-P and IK-G designed the experiments. NS, AK-P, IK-G, and GR performed the experiments. IK-G and NS contributed reagents. IK-G, AK-P, and NS wrote the manuscript. AK-P and GR contributed equally.

### Conflict of interest statement

The authors declare that the research was conducted in the absence of any commercial or financial relationships that could be construed as a potential conflict of interest.
