# Supplementary material for: Collective Vortex-Like Movement of Bacillus subtilis Facilitates the Generation of Floating Biofilms
Source: Front Microbiol. 2018 Mar 29;9:590. doi: 10.3389/fmicb.2018.00590 (PMC5884953; doi:10.3389/fmicb.2018.00590)
Supplement: Supplementary file 2 [file Presentation1.PDF]

Supporting Material

Supplemental Video Legends

Video S1– Top-view of pellicle formation time-lapse images of WT and motility and chemotaxis strains

Pellicle development assay was performed as described in Experimental Procedures, in 48 well plates at room temperature. Photo images were taken every 15 min for 50 hours, and compressed to a 10-frames-per-second video. Time of each frame is indicated at the top right corner. Scale bars represent 2 mm.

Video S2– Side-view of pellicle formation time-lapse images of WT and motility and chemotaxis strains

Pellicle development assay was performed as described in Methods, in 48 well plates at room temperature. Photo images were taken every 15 min for 50 hours, and compressed to a 10-frames-per-second video. Time of each frame is indicated at the top right corner. Scale bars represent 2 mm.

Figure S1– Top-view of mature pellicles formed by extracellular matrix mutants

Pellicle development assay was performed as described in Experimental Procedures, in 6 well plates at 23°C. Photo images were after 40 hours.

Figure S2– Top-view of macro aggregates formed by motility mutants prior to pellicle formation

Pellicle development assay was performed as described in Experimental Procedures, in 6 well plates at 23°C. Photo images were after 18 hours.

Figure S3– Re-aggregation following interference during pellicle formation. Shown is a pellicle formation time in the wild-type and its *tasA* mutant derivative in standing liquid cultures in six well plates at 23°C after 20 hours of incubation. At either 10 hours (v1), or 10 and 16 hours (v2) and 10, 16 and 20 hours (v3) post inoculation the growth

medium was collected, robustly vortexed and returned to the well. The delay in pellicle formation was the difference between un-vortexed controls within the same experiment, and vortexed samples. Results are an average and standard deviation of 3 independent repeats performed in duplicates.

.

Figure S1

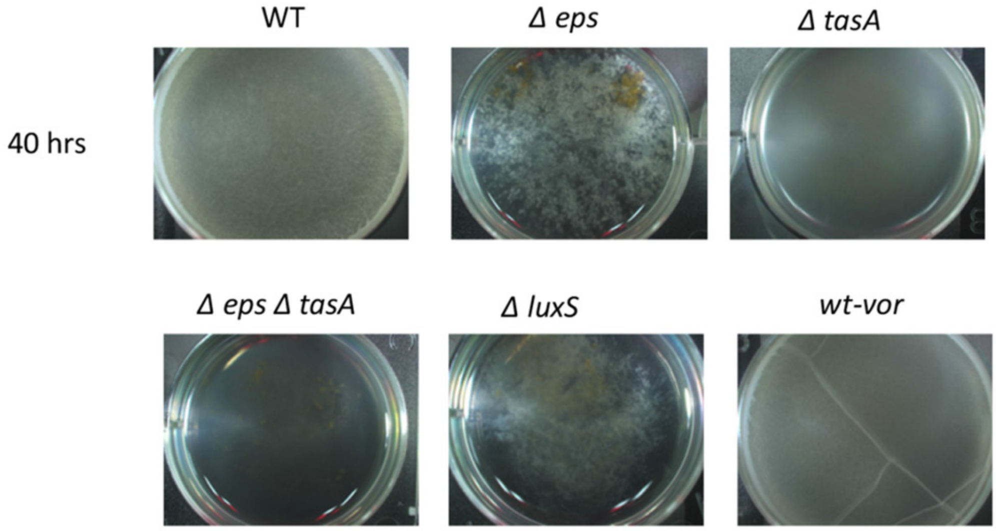

Figure S2

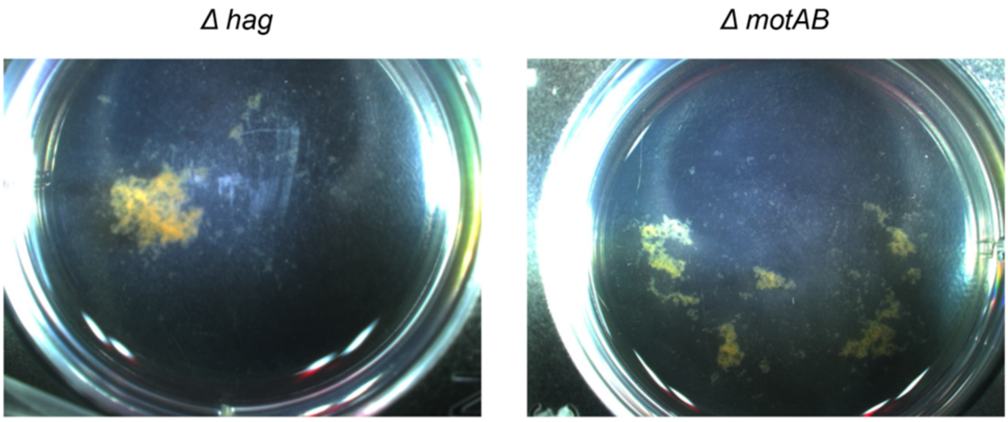

Figure S3

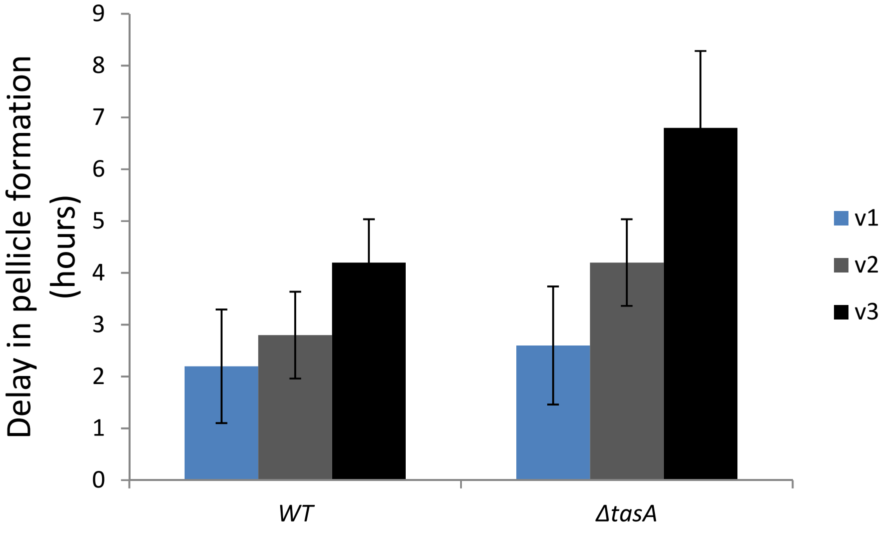

Delay in pellicle formation (hours)
9
8
7
6
5
4
3
2
1
0
WT
ΔtasA
v1
v2
v3

Supplemental References

Blair, K.M., Turner, L., Winkelman, J.T., Berg, H.C., and Kearns, D.B. (2008). A molecular clutch disables flagella in the Bacillus subtilis biofilm. Science *320*, 1636-1638.

Branda, S.S., Chu, F., Kearns, D.B., Losick, R., and Kolter, R. (2006). A major protein component of the Bacillus subtilis biofilm matrix. Molecular microbiology *59*, 1229-1238.

Branda, S.S., Gonzalez-Pastor, J.E., Ben-Yehuda, S., Losick, R., and Kolter, R. (2001). Fruiting body formation by Bacillus subtilis. Proc Natl Acad Sci U S A *98*, 11621-11626.

Branda, S.S., Gonzalez-Pastor, J.E., Dervyn, E., Ehrlich, S.D., Losick, R., and Kolter, R. (2004). Genes involved in formation of structured multicellular communities by Bacillus subtilis. Journal of bacteriology *186*, 3970-3979.

Chu, F., Kearns, D.B., Branda, S.S., Kolter, R., and Losick, R. (2006). Targets of the master regulator of biofilm formation in Bacillus subtilis. Molecular microbiology *59*, 1216-1228.

Kearns, D.B., and Losick, R. (2003). Swarming motility in undomesticated Bacillus subtilis. Mol Microbiol *49*, 581-590.

Vlamakis, H., Aguilar, C., Losick, R., and Kolter, R. (2008). Control of cell fate by the formation of an architecturally complex bacterial community. Genes & development *22*, 945-953.
